# Incidental Popliteal Bursa Localization on I-131 Whole-Body Scintigraphy

**DOI:** 10.3390/diagnostics16203431

**Published:** 2026-10-21

**Authors:** Jocelyn Chu, Wai Han Ma

**Affiliations:** Department of Radiology and Nuclear Medicine, Tuen Mun Hospital, 23 Tsing Chung Koon Rd, Tuen Mun, Hong Kong, China; mwh466@ha.org.hk

**Keywords:** popliteal bursa, Baker’s cyst, I-131 scintigraphy, false-positive iodine uptake

## Abstract

I-131 scintigraphy can be performed after I-131 radioactive iodine (RAI) treatment in appropriate patients for dynamic risk stratification. Sodium iodide symporter (NIS) expression is a mechanism responsible for uptake of I-131 in thyroid tissue and thyroid cancer. There are other mechanisms that iodine engages in the body, leading to possible false-positive findings. A popliteal bursa, or Baker’s cyst, is a bursa that lies in the popliteal region. As a bursa, it is a potential space and is lined by synovial membrane. It is not normally visualized on imaging. However, passive diffusion of radioactive iodine or pathological changes occur with local irritation. Presented herein is a 75-year-old woman with a history of poorly differentiated thyroid carcinoma. Her postoperative American Thyroid Association risk stratification was high. A diagnostic iodine whole body scintigraphy for dynamic risk stratification following RAI treatment performed. Planar imaging on posterior-view showed mildly intense iodine uptake in the left knee, differentials included atypical location of osseous metastasis, and false-positive pathologies. Regional SPECT/CT localized the false-positive iodine uptake in the Baker’s cyst. The teaching point for this case is to be vigilant of uncommon false-positive pathologies on iodine scintigraphy. Diagnostic confidence is improved by adding SPECT/CT imaging.

**Figure 1:**
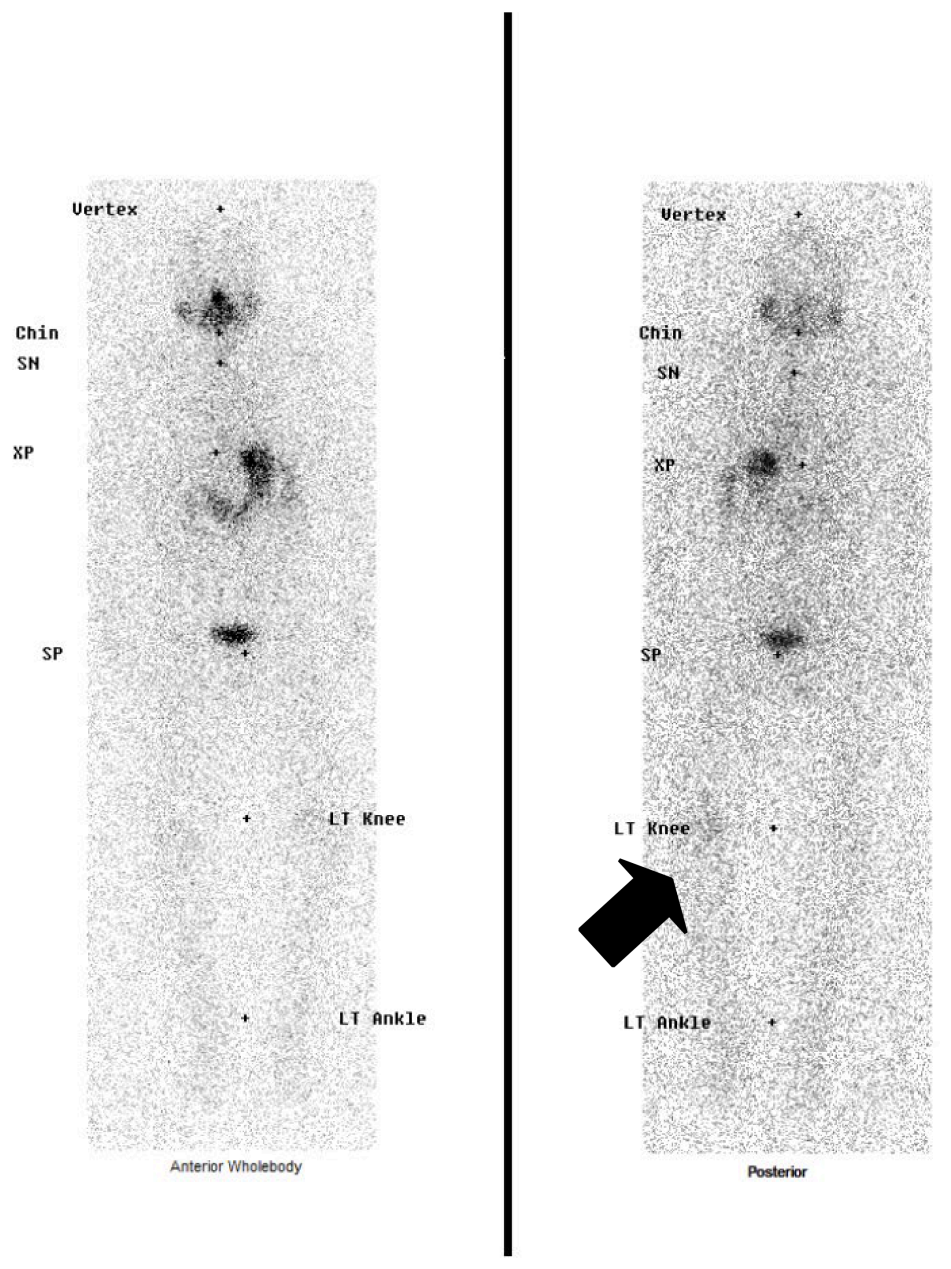
Diagnostic I-131 whole-body scan shown in anterior and posterior views [1]. Each view is presented in parallel with its respective anatomical marker reference points. Curvilinear uptake seen on posterior view at the level of the left knee is shown (black arrow) [2,3,4]. Physiological iodine uptake seen in the nasal mucosa, salivary glands, gastrointestinal tract, and urinary bladder is also shown. Vertex: vertex of skull; SN: sternal notch; XP: xyphoid process; SP: suprapubic; LT: left; RT: right.

**Figure 2:**
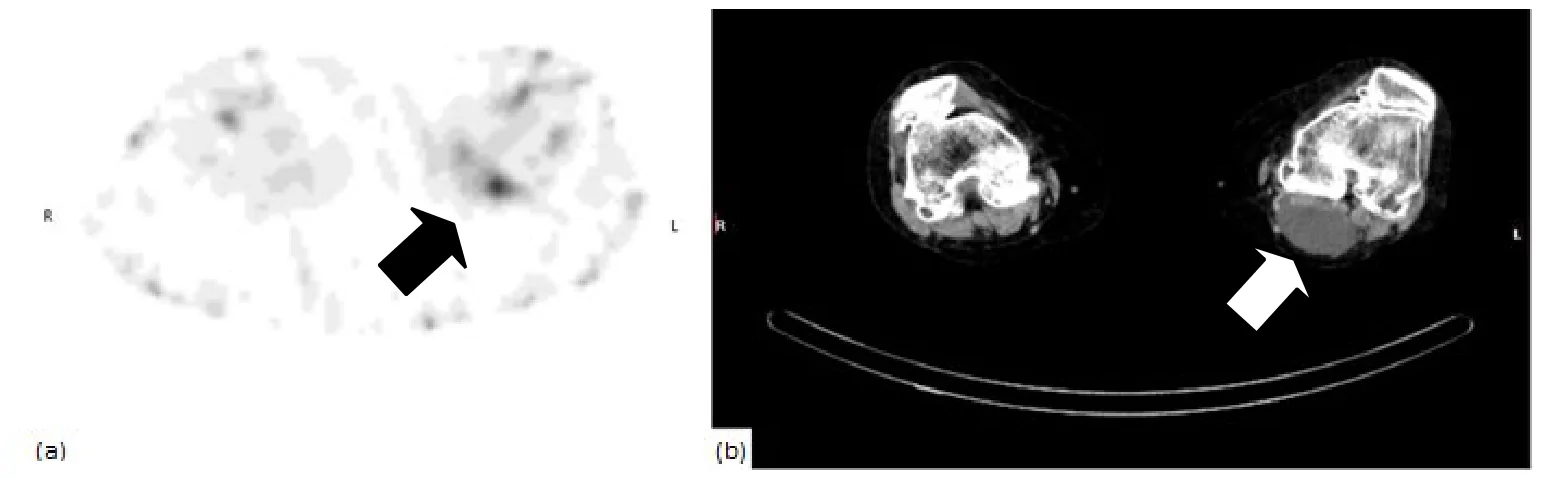
(**a**) Single-photon emission computerized tomography (SPECT) image demonstrating (black arrow) increased uptake in the left posterior knee . L: left; R: right. (**b**) Concurrently acquired computerized tomography (CT) image localizing the iodine uptake to the left Baker’s cyst (white arrow), measuring 6.4 × 3.3 × 6.2 cm. L: left; R: right.

**Figure 3:**
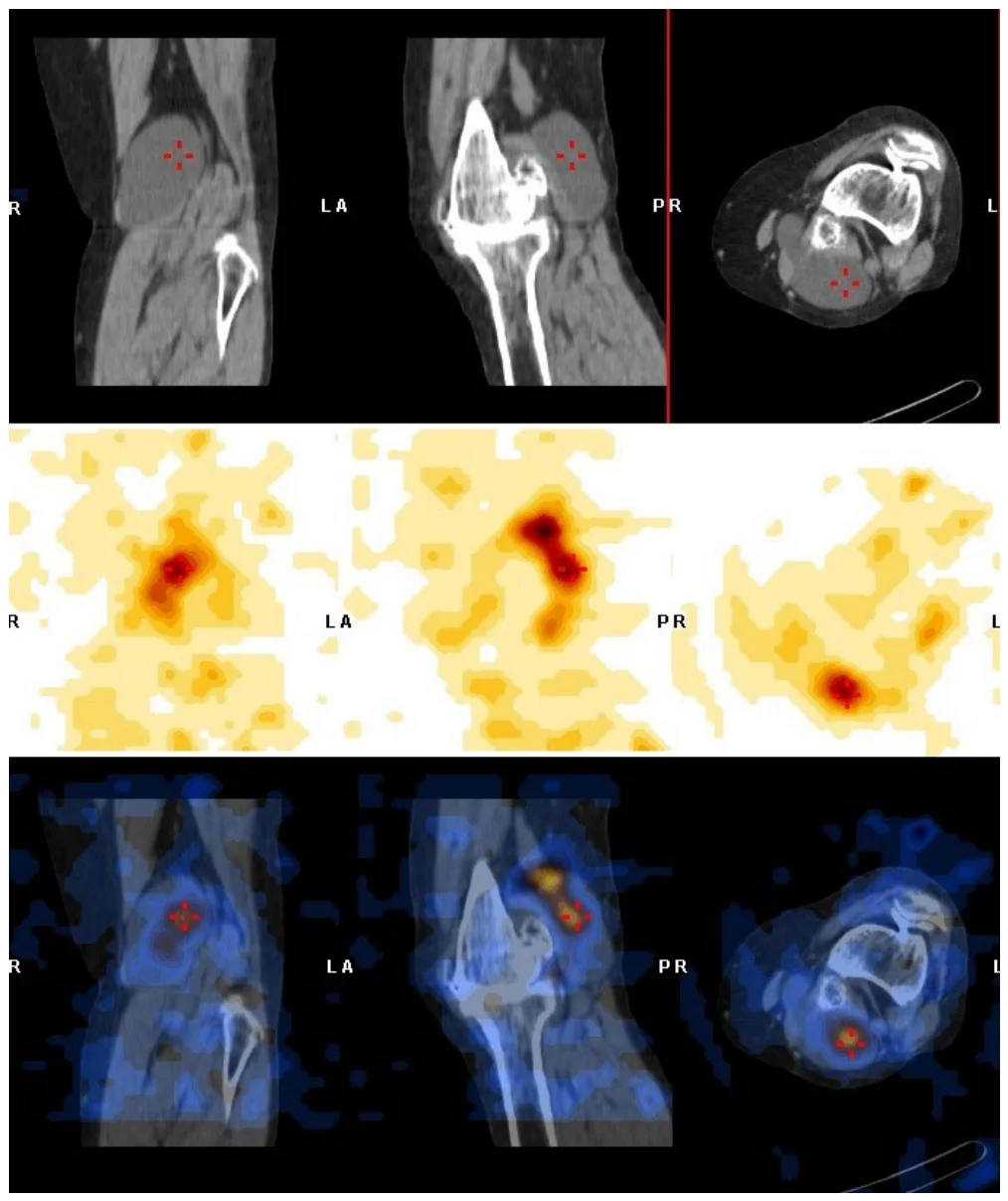
SPECT/CT of the left knee identifying the iodine-avid left-knee Baker’s cyst (cross arrow). Presented in CT, SPECT, and SPECT/CT fusion images in axial, sagittal, and coronal views. R: right; L: left; A: anterior; P: posterior.

## Data Availability

Data is unavailable due to ethical restrictions.

## Abbreviations

The following abbreviations are used in this manuscript:

I-131	Iodine 131
SPECT/CT	Single-photon emission computerized tomography/computerized tomography
RAI	Radioactive iodine
Tg	Thyroglobulin
TSH	Thyroid-stimulating hormone
Anti-Tg	Anti-thyroglobulin antibody
